# Berberine regulates the protein expression of multiple tumorigenesis-related genes in hepatocellular carcinoma cell lines

**DOI:** 10.1186/s12935-017-0429-3

**Published:** 2017-05-30

**Authors:** Tung-Yueh Chuang, Hsiao-Li Wu, Jie Min, Michael Diamond, Ricardo Azziz, Yen-Hao Chen

**Affiliations:** 1 Department of Obstetrics/Gynecology, Augusta University, 1120 15th Street, CA-2020, Augusta, GA 30912 USA; 2 Department of Medicine, Medical College of Georgia, Augusta University, Augusta, GA 30912 USA; 3 Department of Obstetrics and Gynecology, Union Hospital, Tongji Medical College, Huazhong University of Science and Technology, Wuhan, China

**Keywords:** Berberine, Hepatocellular carcinoma, KLF6, ATF3, p21, E2F1, PTTG1, ERK1/2

## Abstract

**Background:**

Hepatocellular carcinoma (HCC) is the seventh most common malignancy and the third leading cause of cancer-related death worldwide with an extremely grim prognosis. Berberine (BBR) has been found to inhibit proliferation of human HCC cells, although the underlying mechanism(s) are unclear.

**Methods:**

Protein expression was detected by Western blots. Cell viability was determined by using the CellTiter Assay kit.

**Results:**

We confirm that BBR treatment inhibits HepG2, Hep3B, and SNU-182 cell viability, and suggest that it regulates this proliferation via the modulation of multiple tumorigenesis-related genes protein expression. BBR treatment up-regulated protein expression of tumor suppressor genes, including Kruppel-like factor 6 (KLF6), activating transcription factor 3 (ATF3) and p21, while down-regulating the expression of selected oncogenes, including E2F transcription factor 1 (E2F1) and pituitary tumor transforming gene 1 (PTTG1). The specific extracellular signal–regulated kinases 1/2 (ERK1/2) inhibitor, PD98059, partially inhibited BBR effects including reduction of cell viability, and up-regulation of KLF6 and ATF3 expressions; although, PD98059 did not alter the down-regulation of E2F1 and PTTG1 expression by BBR.

**Conclusions:**

Our results suggest that BBR inhibits HCC cell viability by modulating multiple tumorigenesis-related genes, and that up-regulation of tumor suppressor genes by BBR is in part the result of ERK1/2 action. The results of this study augment our understanding of the mechanisms underlying the effect of BBR on hepatocellular cancers and provide further evidence as to the biological plausibility of this agent’s role in the treatment of these malignancies.

**Electronic supplementary material:**

The online version of this article (doi:10.1186/s12935-017-0429-3) contains supplementary material, which is available to authorized users.

## Background

Hepatocellular carcinoma (HCC) is the seventh most common cancer and the third leading cause of cancer death worldwide, with few therapeutic options [1]. The American Cancer Society indicates that there are more than 600,000 deaths and over 700,000 new cases of primary liver cancer in the world each year [2]. The treatment of HCC continues to be a challenge; the outcome of traditional surgical treatment is poor with 20% survival at 1 year, 5% at 3 years, and a median survival of 8 months [3]. Although chemotherapy is of considerable benefit to patients with HCC, it is associated with significant side-effects; hence, highlighting the need for therapeutic strategies that target tumor cells without causing cytotoxicity in healthy hepatocytes [4, 5].

Berberine (BBR) is an isoquinoline alkaloid, which can be isolated from a variety of naturally occurring plants such as *Coptidis rhizoma*, *Phellodendron chinense schneid*, and *Phellodendron amurense* [6]. BBR has an anti-tumor effect on many cancers including melanoma, neuroblastoma, lung cancer, colonic carcinoma, breast cancer, and HCC [7–12]. BBR acts both in vitro and in vivo to suppress human cancer cell growth via suppression of tumor cell proliferation, induction of tumor cell apoptosis, and inhibition of both invasion and metastasis [6, 13]. In HCC, BBR inhibits proliferation and migration as well as induces cell cycle arrest and apoptosis [8, 14–19]. However, BBR demonstrates very low to no cytotoxic effect on healthy liver tissue [18]. In addition, BBR appears to have a protective effect on healthy liver tissue specifically protective against chemically-induced hepatotoxicity [20].

Many tumor suppressor genes and oncogenes are related to HCC tumorigenesis. Expression of tumor suppressor genes including Kruppel-like factor 6 (KLF6) [21, 22], activating transcription factor 3 (ATF3) [23], and the cyclin-dependent kinase inhibitor protein *p21* [24] have been found to be reduced in HCC. KLF6 is down-regulated in several types of cancers, and overexpression of wild-type KLF6 inhibits HCC cells proliferation and migration [21, 25, 26], while KLF6 down-regulation by siRNA increases HepG2 proliferation [22]. ATF3 promotes cell death, cell arrest and suppresses Ras-mediated tumorigenesis [27]. In HCC, Niclosamide induces cell apoptosis via upregulation of ATF3 and activation of pERK [28]. p21 has been found to inhibit DNA synthesis and proliferation in human liver cancer cells [24, 29]. Alternatively, oncogenes pituitary tumor transforming gene 1 (PTTG1) [22, 30, 31] and E2F transcription factor 1 (E2F1) [32] are overexpressed in HCC. Reduced expression of PTTG1 decreases cell proliferation and induces apoptosis in HCC cells [22, 31], while overexpression of E2F1 promotes HCC cell growth and invasion [33].

The above findings suggest that BBR is a promising candidate for the treatment of HCC. However, the molecular mechanisms underlying the anti-neoplastic action of BBR in HCC are not fully understood. It is possible that BBR acts by modulating these tumor suppressor genes and oncogenes known to play a role in HCC, a hypothesis tested in the present study.

## Methods

### HCC cell lines

HepG2 (Cat# HB-8065), Hep3B (Cat# HB-8064) and SNU-182 (Cat# CRL-2235) were purchased from ATCC (Manassas, VA, USA). HepG2 and Hep3B cells were cultured in medium Dulbecco’s Minimum Essential Medium (DMEM) supplemented with 10% fetal bovine serum (FBS). SNU-182 cells were cultured in medium RPMI 1640 with 10% FBS. All cells were cultured at 37 °C in a humidified chamber with 95% air and 5% CO_2_.

### Chemicals

BBR (Cat# B3251) and PD98059 (Cat# sc-3532) were purchased from Sigma-Aldrich (St. Louis, MO, USA) and Santa Cruz Biotechnology (Dallas, TX, USA). The concentration of stocks are 10 mM in water for BBR and 25 mM in DMSO (Sigma-Aldrich, St. Louis, MO, USA) for PD98059.

### Western blots

Cells were seeded in 12 wells plate with cell number 1 × 10^5^ per well overnight and then treated PD98059, BBR or both for 24 h. After treated, samples were lysed with a lysis buffer, and protein concentrations were determined by using coomassie blue method. Forty µg of total protein were separated on SDS-PAGE gels and then transferred to PVDF membranes. Membranes were immunoblotted with the appropriate primary antibodies (KLF6, ATF3, p21, E2F1, PTTG1, total ERK1/2, and phosphor-ERK1/2) (Santa Cruz, Dallas, TX, USA) at 4 °C overnight. After washing, membranes were incubated with a secondary antibody (Jackson ImmunoResearch Laboratory, West Grove, PA, USA), detected with chemiluminescence reagent (Thermoscientific, Hampton, NH, USA) and exposed by autoradiography.

### Cell viability assay

Cell viability was determined by using the CellTiter Assay (MTS) kit (Promega, Madison, WI, USA). Cells were trypsinized and seeded 5000 cell/well into 96-well plates and incubated overnight in DMEM with 10% FBS in CO_2_ incubator. After overnight incubation, cells were treated with or without BBR for 24 or 72 h in DMEM with 5% FBS. Prior to conducting the cell viability assay, cells were washed with PBS twice and incubated in PBS 100 μl/well. Twenty microliters of CellTiter solution was added to each well. Cells were incubated in CO_2_ incubator for 2 h. Absorbance was determined with a microplate reader at 490 nm.

### Cell number count

Cells were trypsinized and seeded 40,000 cells/well into 12-well plates and incubated overnight in DMEM with 10% FBS in CO2 incubator. After overnight incubation, cells were treated with or without BBR for 72 h in DMEM with 5% FBS. After 72 h treatment, cells were trypsinized, and Cell number were counted by the hemocytometer under a microscope. Dead cells were excluded by Trypan Blue stain.

### ERK1/2 stimulation and PD98059 experiments

Cells were pretreated with dimethyl sulfoxide (DMSO, solvent of PD98059 used as a control) or PD98059 25 µM for 30 min then treated with or without BBR 100 µM for 24 h. Cell viability was analyzed by MTS assay. ERK1/2 phosphorylation, and BBR- regulated protein expression was analyzed by western blot.

### Statistical analysis

Comparisons of multiple groups were carried out by analysis of variance (ANOVA), followed by a post-test using the Fisher (among groups) or Dunnett (compared with control group) tests (XLSTAT Software, New York, NY, USA). A *p* < 0.05 was considered statistically significant. All experiments were repeated 3 times (n = 3). All values are presented as mean ± SEM.

## Results

### BBR inhibits HCC cells viability in a time and dose-dependent manner

**Fig. 1:**
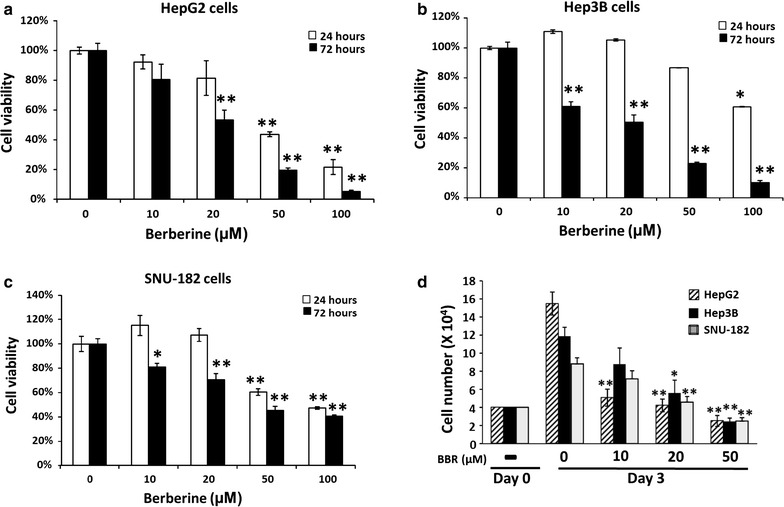
BBR inhibits cell viability and proliferation. **a** HepG2, **b** Hep3B and **c** SNU-182 cells were treated with various doses of BBR (0, 10, 20, 50, and 100 μM) for 24 and 72 h. Cells viability were detected by MTS assay. **d** Cell numbers were counted after treated with various doses of BBR (0, 10, 20, 50) for 72 h. All experiments were repeated 3 times (n = 3). All data are depicted as mean ± SEM (*error bars*), (*p < 0.05; **p < 0.01 vs. BBR 0 μM)

Three HCC cell lines were treated with BBR in concentrations of 0, 10, 20, 50 and 100 µM for 24 and 72 h. Cell viability was detected by MTS assay. After 24 h of treatment, BBR in concentrations of 50 and 100 μM inhibited HepG2 cell viability approximately 50 and 80% respectively, and BBR in concentrations of 20, 50 and 100 μM inhibited HepG2 cell viability approximately 40, 80 and 95%, respectively with 72 h treatment (Fig. 1a). In Hep3B cells with 24 h treatment, BBR concentrations of 100 μM inhibited cell viability approximately 40%, and, BBR concentrations of 10, 20, 50 and 100 μM inhibited Hep3B cells viability approximately 40, 50, 80 and 90%, respectively with 72 h treatment (Fig. 1b). Treated SNU-182 cells with BBR concentrations of 50 and 100 μM for 24 h inhibited cell viability approximately 40 and 50%, respectively. 72 h treatment, with BBR in concentrations of 10, 20, 50 and 100 μM inhibited SNU-182 cell viability approximately 20, 30, 50 and 60%, respectively (Fig. 1c). To confirm that BBR inhibits HCC cells proliferation, cell numbers were counted after BBR treated for 3 days. HCC cell numbers were all increased after 72 h culture, and BBR was able to inhibit HCC cells proliferation (Fig. 1d). BBR in the concentration of 10 µM with 72 h treatment significantly inhibited HepG2 cells proliferation approximately 68% but not in Hep3B or SNU-182 cells. BBR in the concentration of 20 µM significantly inhibited HepG2, Hep3B and SNU-182 cells proliferation approximately 72, 50 and 50%, respectively. Overall, BBR inhibited HCC cell viability and proliferation in a time and dose-dependent manner; however, different cell lines show varying degrees of sensitivity to BBR treatment. HepG2 cells were the most sensitive to BBR treatment within these three HCC cell lines.

### BBR regulates protein expression of tumor suppressor genes and oncogenes in HCC

**Fig. 2:**
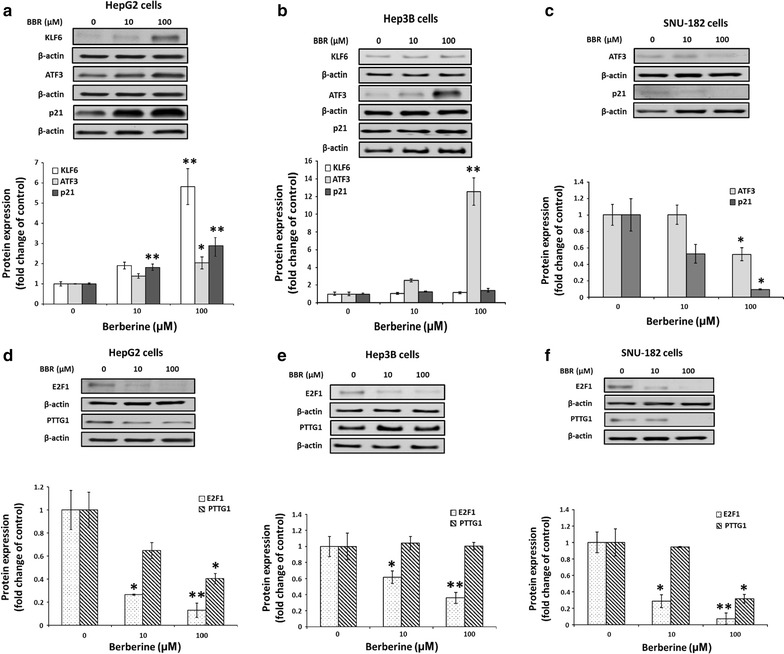
BBR regulates protein expression of tumor suppressor genes and oncogenes. Cells were treated with various doses of BBR (0, 10, and 100 μM) for 24 h. **a**, **d** HepG2, **b**, **e** Hep3B and **c**, **f** SNU-182. All experiments were repeated 3 times (n = 3). All data are depicted as mean ± SEM (*error bars*), (*p < 0.05; **p < 0.01 vs. BBR 0 μM)

Cells were treated with BBR in concentrations of 0, 10 and 100 μM for 24 h and protein levels of KLF6, ATF3 and p21 were determined. BBR dose dependently stimulated KLF6 (p < 0.01), ATF3 (p < 0.05) and p21 (p < 0.05) expression with stimulation reaching significance at a concentration of 100 μM in HepG2 cells (Fig. 2a). BBR did not stimulate KLF6 and p21 protein expression in Hep3B cells, but significantly induced ATF3 (p < 0.01) at the highest dose (Fig. 2b). In contrast to HepG2 and Hep3B cells, BBR did not stimulate but inhibited ATF3 (p < 0.05) and p21 (p < 0.05) expression in SNU cells at the highest dose (Fig. 2c). Whether BBR altered KLF6 expression in SNU-182 cells were unable to assess because the KLF6 antibody did not detect KLF6 in SNU-182 cell western blot.

For the oncogenes studied, BBR dose dependently and significantly decreased E2F1 (p < 0.01) and PTTG1 (p < 0.05) expression in HepG2 cells (Fig. 2d). In Hep3B cells, BBR reduced expression of E2F1 (p < 0.01). However, BBR did not alter PTTG1 expression in Hep3B cells (Fig. 2e). A similar response to BBR like that observed with HepG2 was noted, BBR dose dependently and significantly decreased E2F1 (p < 0.01) and PTTG1 (p < 0.05) expression in SNU-182 cells (Fig. 2f).

### BBR activates ERK1/2 phosphorylation, and ERK1/2 specific inhibitor PD98059 partially blocks action of BBR on cell proliferation and protein expression

**Fig. 3:**
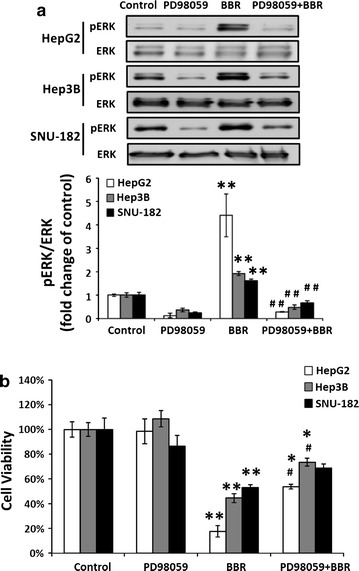
PD98059 (25 µM) inhibits spontaneous and BBR (100 µM) -induced ERK phorphorylation (**a**), and partially blocks BBR-inhibited cell proliferation (**b**). All experiments were repeated 3 times (n = 3). All data are shown as mean ± SEM (*error bars*), (*p < 0.05; **p < 0.01 vs control), (^#^ *p* < 0.05 vs. BBR 100 μM)

To determine whether activation of ERK1/2 played a role in the mechanisms underlying the action of BBR on cell proliferation, tumor suppressor genes and oncogenes expression, All three kind of cells were treated with BBR (100 μM), with PD98059 (25 μM) alone, or with PD98059 in combination with BBR, for 24 h. Treatment of PD98059 inhibited endogenous and BBR-stimulated ERK1/2 phosphorylation (Fig. 3a).

**Fig. 4:**
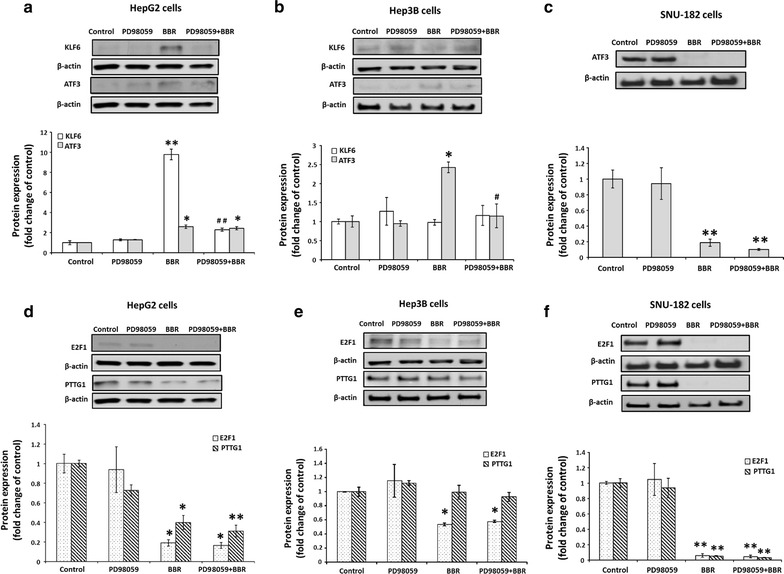
PD98059 (25 µM) partially blocks BBR (100 µM)—regulated protein expression of tumor suppressor genes and oncogenes. **a**, **d** HepG2, **b**, **e** Hep3B and **c**, **f** SNU-182. All experiments were repeated 3 times (n = 3). All data are shown as mean ± SEM (*error bars*), (*p < 0.05; **p < 0.01 vs. control), (^#^ *p* < 0.05, ^##^ *p* < 0.01 vs BBR 100 μM)

PD98059 was able to partially, but significantly (p < 0.05) reduce the inhibition of cell viability by BBR (Fig. 3b); however, this was only observed in HepG2 and Hep3B cells. In turns of BBR-regulated genes, PD98059 completely inhibited BBR-induced KLF6 protein expression (p < 0.01), but had no any effect on BBR-induced ATF3 protein expression in HepG2 cells (Fig. 4a). In Hep3B, PD98059 completely blocked BBR-induced ATF3 protein expression (p < 0.05) (Fig. 4b). PD98059 had no effect on BBR-reduced ATF3 protein expression in SNU cells (Fig. 4c). Whether PD98059 altered BBR-induced p21 protein expression were unable to assess as DMSO, the solvent used for PD98059, was dose dependently stimulated p21 while has no effect on KLF6 protein expression in HepG2 cells (Additional file 1: Figure S1, line 1, 3, 5). In addition, BBR induced p21 protein expression in Fig. 2 and Additional file 1: Figure S1 (line 1 and 2), however combined DMSO with BBR reduced p21 protein expression (Additional file 1: Figure S1, compared line 1 & 2, 3 & 4 and 5 & 6).

PD98059 did not alter BBR-reduced PTTG1and E2F1 expression in HepG2 (Fig. 4d), Hep3B (Fig. 4e) nor SNU-182 (Fig. 4f).

## Discussion

Overall, we confirmed that BBR inhibited HepG2 and Hep3B cell proliferation [17, 18, 34]. In addition, we demonstrated that BBR inhibited cell proliferation of SNU-182 cells. However, HepG2 cells appeared to be the most sensitive, while SNU-182 was the least sensitive to BBR treatment. For example, 100 µM of BBR treatment for 24 h inhibited cell viability by approximately 80% in HepG2 cells, while the same concentration and duration of BBR inhibited cell viability by about 40 and 50% in Hep3B and SNU-182 cells, respectively. In literature, cancer cell line with p53 gene deleted was reported to be more resistant to drug treatment [35]. Hep3B is a p53 deficient cell line, thus it is not surprised that Hep3B is more resistance to BBR treatment than HepG2. This finding is consistent with previously reported results [34]. In addition, HepG2 and Hep3B are “well-differentiated”, while SNU-182 is a “poorly-differentiated” HCC cell line [36]. Our results suggest that “poorly-differentiated” HCC cells is less sensitive to BBR treatment.

In addition to cell proliferation, BBR also regulates gene expression differently between these three HCC cell lines. BBR stimulated expression of three tumor suppressor genes, KLF6, ATF3 and p21, and reduced two oncogenes E2F1 and PTTG1 in HepG2 cells, while BBR just induced ATF3 and reduced E2F1 expression in Hep3B cells. As HepG2 expresses wild type p53 and Hep3B is a p53-deficient HCC cell line, these results suggest that BBR regulation of KLF6, p21 and PTTG1 expression is possibly p53 dependent. Indeed, BBR has been found to up-regulate miR-23a via regulation of p53 [37]. In contrast, BBR reduced expression of ATF3 tumor suppressor genes and also oncogenes E2F1 and PTTG1 in SNU-182 cells. BBR regulated different genes in SNU-182 as compared to HepG2 and Hep3B cells. This difference may be explained by SNU-182’s poorly-differentiated cell line. However, this discordance i.e., the effect of BBR on tumor suppressor genes and oncogenes expression, may also explain the observed differences in the response of cell proliferation to BBR between cell lines.

The ERK1/2-specific inhibitor, PD98059 partially blocked BBR-induced inhibition of cell proliferation in HCC cell lines, suggesting that activation of the ERK1/2 pathway is involved in BBR-inhibited cell proliferation. Indeed, Aspafilioside B, a steroidal saponin extracted from Asparagus filicinus and a known active cytotoxic component, has been shown to induce apoptosis via ERK1/2 activation in HepG2 cells [38]. In addition, PD98059 completely blocked BBR-induced KLF6 and ATF3 expression in HepG2 and Hep3B cells, respectively suggesting that activation of the ERK1/2 pathway is involved in BBR’s-regulation of gene expression in the HCC cell line. However, PD98059 did not block BBR-reduced E2F1 and PTTG1 expression, suggesting that BBR mediated regulation of E2F1 and PTTG1 are independent of ERK1/2 pathway. These results also indicate that ERK1/2 is not the only signaling pathway under BBR regulation.

DMSO, the solvent for PD98059, has been discovered to induce p21 expression in B cell lines [39]. When we did PD98059 experiments, we noticed that DMSO alone also increased p21 protein expression in HepG2. Furthermore, our data indicated that DMSO reversed the effects of BBR on p21 protein expression from stimulation became inhibition. Our data suggest that DMSO effects and interaction with BBR may need to be considerate when doing experiments that are involved DMSO and BBR.

KLF6 has been reported to upregulate p21 [40] and ATF3 [41], but suppress PTTG1 [22] expression in cancer cells. However, in our experiments, PD98059 completely blocked BBR-induced KLF6 expression, but did not block BBR-regulated ATF3 and PTTG1 expression in HepG2 cells. These results suggest that BBR-regulated ATF3 and PTTG1expression was not through KLF6 regulation.

## Conclusions

In conclusion, overall BBR inhibits cell proliferation of tested three HCC cell lines including HepG2, Hep3B and SNU-182. However, BBR-regulated protein expression of multiple tumorigenesis associated genes in these cell lines differently (summary in Table 1) indicated different regulation mechanisms of BBR in these cells. As ERK1/2 inhibition was unable to completely block the effects of BBR, our data suggest that other pathways may also be involved in this regulation. The results of this study augment our understanding of the mechanisms underlying the effect of BBR on hepatocellular cancers and provide further evidence as to the biological plausibility of this agent’s role in the treatment of these malignancies.

**Table 1:** Summary of BBR-regulated protein expression of multiple genes in HCC cell lines

HCC cell lines	Tumor suppress genes			Oncogenes	
	KLF6	ATF3	P21	E2F1	PTTG1
HepG2	+	+	+	−	−
Hep3B	NS	+	NS	**−**	NS
SNU-182	ND	−	−	**−**	−

+, up-regulated; −, down-regulated; NS, non-significant change; ND, non-detectable by western blot

## Additional file

**Additional file 1:** **Figure S1**. DMSO dose dependently regulates p21 protein expression in HepG2 cells. Lines 1 & 2: water; lines 3 & 4: DMSO 0.25 μl/ml and lines 5 & 6: DMSO 0.5 μl/ml.

## Abbreviations

BBR: berberine
HCC: hepatocellular carcinoma
KLF6: Kruppel-like factor 6
ATF3: activating transcription factor 3
E2F1: E2F transcription factor 1
PTTG1: pituitary tumor transforming gene 1
ERK1/2: extracellular signal–regulated kinases 1/2
