# Supplementary figures and images for: Berberine regulates the protein expression of multiple tumorigenesis-related genes in hepatocellular carcinoma cell lines
Source: Cancer Cell Int. 2017 May 30;17:59. doi: 10.1186/s12935-017-0429-3 (PMC5450260; doi:10.1186/s12935-017-0429-3)

**Figure S1.**


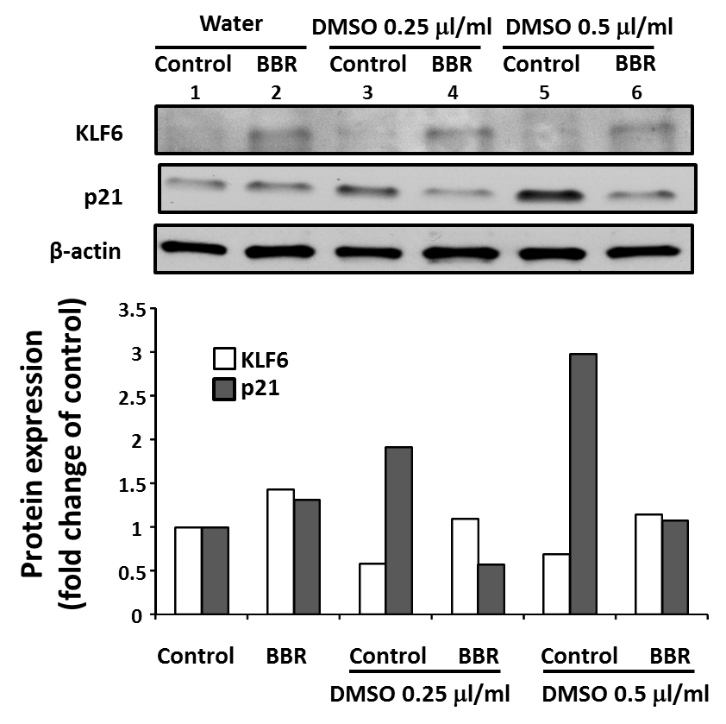

Supplement: Supplementary file 1 — Additional file 1: Figure S1. DMSO dose dependently regulates p21 protein expression in HepG2 cells. Lines 1 & 2: water; lines 3 & 4: DMSO 0.25 μl/ml and lines 5 & 6: DMSO 0.5 μl/ml. [file 12935_2017_429_MOESM1_ESM.docx]
